# Structural Study of Selectivity Mechanisms for JNK3 and p38α with Indazole Scaffold Probing Compounds

**DOI:** 10.21203/rs.3.rs-4730282/v1

**Published:** 2024-08-07

**Authors:** HaJeung Park, Yangbo Feng

**Affiliations:** X-ray Crystallography Core, UF Scripps Biomedical Research; University of Miami

## Abstract

Selectivity is a primary focus in medicinal chemistry for ATP-competitive kinase inhibitors due to the highly conserved ATP binding pockets in the kinome. A decade of medicinal chemistry efforts has been carried out to develop selective inhibitors for JNKs, resulting in the identification of numerous promising scaffolds that even exhibit isoform selectivity. Thiophene-indazole is one of the scaffolds explored for isoform selectivity. Some iterations of this scaffold have also shown selectivity for p38α. In this study, we utilized four compounds derived from thiophene-indazole to investigate the mechanisms of selectivity for JNK3 and p38α. We determined crystal structures of the inhibitors bound to either JNK3 or p38α and subjected them to molecular dynamics (MD) simulations to understand the binding mechanism and critical interactions that govern affinity and selectivity for these two important kinases. The findings from this study provides valuable information for improving current lead inhibitors and developing a new generation of JNK3 isoform inhibitors.

## INTRODUCTION

Protein kinases are responsible for the majority of signal transduction events in eukaryotic cells by phosphorylating target proteins. Uncontrolled kinase activities are associated with cancer, metabolic disorders, and neurodegenerative diseases. They are important targets for chemical intervention through the development of inhibitors. The human genome contains approximately 500 protein kinases, representing approximately 2% of the total genome [1]. Developing kinase inhibitors is primarily concerned with selectivity, as the often-targeted ATP binding sites that are ubiquitous among protein kinases are highly conserved. Therefore, developing effective inhibitors relies on accurate understanding of the binding mechanisms of the target that are distinct from the off-target binding. In general, higher selectivity can be achieved by utilizing the type II binding mode, which involves the DFG-out conformation. However, some of the most selective inhibitors are accomplished through the type I binding mode [2].

JNK3 and p38α are two heavily studied targets that have received extensive focus in the development of selective inhibitors. C-Jun terminal kinases (JNKs) are at the final step of the stress response signaling, converging the stress and inflammation signals to downstream transcription factors. Inhibition of the pathway is critical for the treatment of cancer and neurodegenerative disorders [3–5]. JNK3 is expressed restrictively in the brain, heart, and pancreas, while JNK1 and JNK2 are expressed ubiquitously. Like JNK activation, p38 kinases are activated by environmental stressors such as UV radiation, oxidative stress, osmotic stress, and inflammatory signals [6]. Out of the four isoforms, p38α is expressed abundantly in most cell types. JNK3 and p38α have a sequence identity of 48%, and the structures of the ATP analog bound states overlap with a root mean square deviation (RMSD) of 1.6 Å. Additionally, the ATP binding pockets have an even higher similarity with a sequence identity of 80% [7]. As a result, the selectivity against p38 has been a touchstone in the development of JNK isoform-selective inhibitors. The selectivity of JNK3 over p38α can be attributed to a slightly smaller active site pocket and a larger hydrophobic gatekeeper residue in JNK3. This combination prevents easy access to the hydrophobic site I. The isoform selectivity of JNKs has also been studied previously [7–10]. One of the key findings for selectivity between JNK3 and JNK1 is the change from Leu144 in JNK3 to Ile106 in JNK1 in the hydrophobic pocket I, resulting in unfavorable bindings when compounds clash with the Cβ-methyl of isoleucine in JNK1.

**Figure 1:**
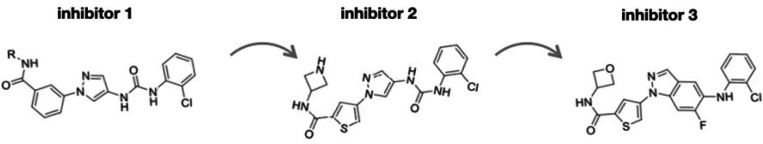
The pyrazole-urea scaffold (Inhibitor 1) and strategies for further optimization led to the development of thiophene pyrazole-urea (Inhibitor 2) and thiophene-indazole (Inhibitor 3).

We have reported several JNK-selective inhibitors with various scaffolds that have demonstrated promising selectivity for JNK over other kinases. Some of these inhibitors have also shown selectivity among the three JNK isoforms (Fig. 1). [7–11]. Our medicinal chemistry campaign aim to enhance the bioavailability of the pyrazole-urea scaffold (inhibitor 1) in JNK3 inhibitor design led to the evolution of the scaffold to the thiophene pyrazole-urea (inhibitor 2) and then to the thiophene-indazole (inhibitor 3) [8,9]. The latest series of inhibitors derived from the thiophene-indazole scaffold exhibited good brain penetration and an excellent selectivity profile [8]. Profiling studies using 370 wild-type kinases have demonstrated that some compounds have distinct selectivity for either JNK3 or p38α, with only subtle alterations.

To investigate and directly compare the selectivity mechanisms between JNK3 and p38α, we have determined X-ray crystal structures of these kinases in complex with four thiophene-indazole compounds. Out of the four compounds, two exhibit selectivity towards JNK3, while the other two demonstrate selectivity towards p38α (Table 1). Molecular dynamics (MD) is a rigorous theoretical tool used to study the relationship between protein structure and function by comparing the characteristics of protein microenvironments using various physicochemical descriptors [12,13]. Therefore, we employed MD simulations in our study to further investigate the interactions between the compounds and their respective kinases. The combination of structural analyses and MD simulations offers a comprehensive understanding of the selectivity mechanism and provides insights for further improvement of the scaffold in developing selective inhibitors for JNK3 isoforms.

## RESULTS

### Overview of JNK3 structures

**Figure 2:**
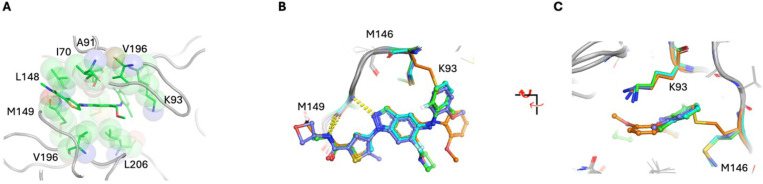
Superposition of JNK3 complexes. A, The interaction of the ligand with the hydrophobic pocket of JNK3 is illustrated using JNK3:21J as the representative example. The key hydrophobic residues are shown as sticks with transparent spheres, and the ligand is shown as sticks. B and C, The conformational differences between JNK3:21M and the rest of the complexes are clearly shown in the overlaps. The side chain of Met146 occupies the void produced by the rotation of the methoxy phenyl ring. The conformation affords cation-π interaction between Lys93 and 21M. The conserved H-bond interactions between the hinge residue, Met149, and the bound inhibitors are indicated with yellow dashed lines. The bound inhibitors and the corresponding residues are colored green, cyan, orange, and blue for 21J, 21G, 23M, and 23GA, respectively.

The JNK3 structures with the four compounds superimpose well with an average RMSD of 0.23 Å over 314 residues. The structural differences arise mainly from the P-loop residues (also known as the glycine-rich loop), where B-factors are elevated compared to the rest of the proteins (**Suppl. Figure 1**). Some residues in the P-loop are untraceable due to poor electron density. A common feature among the structures is that a critical hydrophobic interaction is achieved with the indazole ring where it is stacked between Ala91 from the N-lobe and Val196 and Leu206 from the C-lobe. Additional hydrophobic residues, Ile70, Val78, and Leu148, further augment the hydrophobic pocket (Fig. 2A). Furthermore, the hinge binding geometry is achieved through the thiophene linkage between the amide and indazole, where the nitrogen atom N2 of indazole and the NH group of the amide form H-bonds with the backbone of Met149 (Fig. 2B and 2C). Previously, compounds with an indazole scaffold utilized a phenyl group as the linkage. Our current structures demonstrate that a 5-ring heterocycle, such as thiophene, can offer a hydrogen bond interaction similar to that of the phenyl linker, allowing a noble chemotype design.

Three of the JNK3:compound structures have the side chain of the gatekeeper residue, Met146, pointing away from the binding pocket in order to accommodate the bound compounds to access the hydrophobic pocket I. In the case of JNK3:23M, the methoxyphenyl group swings approximately 90° outward compared to the other compounds. This provides room for the gatekeeper residue to reside in the pocket. The aliphatic side chain of Lys93 typically stacks with an aromatic ring of the hydrophobic group that occupies the hydrophobic pocket I. However, the amine group of Lys93 interacts with the methoxyphenyl through cation-*pi* interaction in the JNK3:23M complex (Fig. 2C). The cause for the methoxyphenyl swing is unclear. The P-loop is relatively stable in JNK3:23M compared to that of JNK3:23GA possibly due to the methyl group of 23GA that is packed against glycine in the P-loop.

### Overview of p38α structures

**Figure 3:**
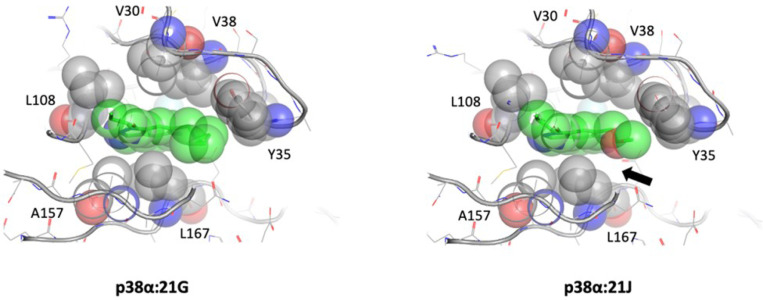
Structural comparison of p38α:21G and p38α:21J at the hydrophobic interface. The N-methyl azetidine substitution at C6 of indazole in 21G demonstrates superior VdW interaction with both Tyr35 and Leu167 compared to the dimethyl ether substitution in 21J. The black arrow indicates the VdW void in the p38α:21J complex. The key residues and the compounds are depicted as sticks with transparent spheres, and the key residues are labeled.

All the p38α structures align well with each other, with a root mean square deviation (RMSD) of 1.1 Å (**Suppl. Figure 2**). The ATP binding pockets as well as the bound inhibitors of the structures also superimpose with the JNK3 structures. Two of the p38α structures show a DFG-in state, while the other two are in a DFG-out state (**Suppl. Figure 2**). In both DFG-out structures, an n-Octyl-β-d-Glucoside (β-OG) occupies the void generated by the rearrangement of the activation loop. Since the crystallization conditions are identical for all four inhibitor-bound structures and the compounds are ‘soaked-in’, we can conclude the DFG-in/-out conformations are induced by the compounds. The β-OG that partially occupies the extended pocket in DFG-out conformation is an opportunistic binder resulting from the loop rearrangement. Similar to the inhibitor bindings observed in JNK3, the hinge binding in p38α is provided by two H-bond interactions between HN and O of the Met109 backbone to N and HN across the thiophene from the compounds. The Thr106, the gatekeeper in p38α, does not influence the binding of the compound, except it creates a slightly hydrophobic environment by positioning the Cβ group towards the pocket. In both 21G and 21J, the addition of a hydrophobic group at the C6 position of the indazoles interacts with Tyr35 in the P-loop, causing the tip of the P-loop to fold towards the ATP binding pocket (Fig. 3). The interaction is augmented by the H-bond between Asp168 and N of the anilino group. There are also one or two β-OG molecules bound in C-lobe at a crevasse generated by helices αEF, α1L14, and α2L14. The implications of these interactions are not well understood [14].

### Detailed analyses of compound bindings

#### JNK3:21G vs. JNK3:21J:

**Figure 4:**
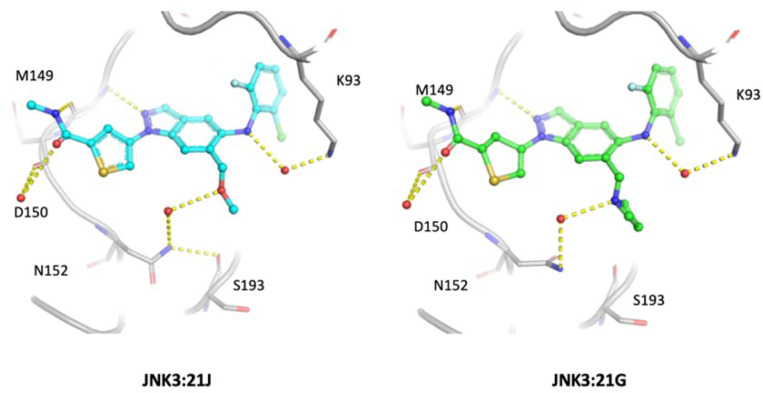
Comparison of the key water-mediated H-bond interactions. Three significant water-mediated H-bond interactions are observed in both JNK3:21J and JNK3:21G. The primary difference lies in the H-bond interaction mediated by Asn152, where the rotation of the side chain is locked in by Ser193.

21J has an enzymatic IC_50_ approximately 23 times lower than 21G. Three water-mediated H-bond interactions are observed between JNK3 and the compounds (Fig. 4). The two water-mediated H-bond interactions by Asp150 and Lys93 to amide oxygen and aminophenyl NH, respectively, were observed previously with the crystal structures of JNK3:pyrazole urea inhibitor complexes [9]. A new water-mediated H-bond interaction is observed between the amide oxygen of Asn152 and the dimethyl ether of 21J (Fig. 4). Furthermore, Asn152 forms an additional H-bond interaction with Ser193, strengthening the overall hydrogen bond network. A similar H-bond interaction is also observed between Asn152 and 21G. However, there is no additional H-bond interaction between Asn152 and Ser193. This is likely because the bulky N-methyl azetidine group disfavors the rotameric state of Asn152 from forming such a H-bond interaction. The N-methyl azetidine group appears to be at a collision distance with the backbone oxygen of Ser193. 0.5 µs MD simulations in the presence of explicit water corroborate with the crystallographic observation. The water-mediated H-bond network between Asn152 and dimethyl ether at 21J was observed 42% of the simulation time. In contrast, no water-mediated interaction between Asn152 and N-methyl azetidine at 21G was observed during the simulation (**Suppl. Figure 3**). The hinge binding interaction and the water-mediated H-bond between Asp150 and amide oxygen are qualitatively similar for both complexes during the simulation time.

#### JNK3:23GA vs. JNK3:23M:

The position of 23GA shifts laterally about 0.5 Å away from the hydrophobic pocket I compared to other compounds. This shift is due to the methyl addition of 23GA at thiophene, which forms a van-der-Waals (VdW) interaction with the side chains of Asn152 and Val196. (Fig. 2B **and Suppl. Figure 4**). During the MD simulation, 23GA intermittently collided with Asn152, causing the compound to shift its position that affected the interaction with the hinge residues. While 23GA still maintained the important hinge binding interaction between Met149 and the indazole nitrogen, the H-bond interaction between Met149 and the amide nitrogen was observed for only 67% of the simulation time for JNK3:23GA. In contrast, the equivalent H-bond interactions in JNK3:23M were more stable and occurred for 95% of the simulation time (**Suppl. Figure 5**). A similar shifting in the bound compound was observed in the crystal structure of the JNK3:aminopyrimidine complex when Leu144 was replaced with the bulkier Ile in the hydrophobic pocket I, which negatively impacted the binding of the inhibitor. Para-substitution of the phenyl group was identified as the crucial factor determining the potency of the aminopyrimidine compounds, determining the selectivity for JNK2/3 over JNK1 [7].

#### p38α:21G vs. p38α:21J:

The P-loop in p38α has been observed to adopt a wide range of conformations in order to accommodate the bound ligand. The conformation is primarily driven by the key residue Tyr35, which is located at the tip of the loop and forms a hydrophobic interaction with the bound ligand. 21G has an advantage over 21J in terms of hydrophobic interaction with Tyr35 due to its substitution at C6 of indazole. Specifically, the N-methyl azetidine group in 21G bordered snuggly against the tyrosyl group, while the end methyl group of dimethyl ether in 21J forms a weak hydrophobic interaction with the tyrosyl side chain of Tyr35. Additionally, the N-methyl azetidine in 21G forms a tighter VdW interaction with Leu167, which is located opposite to Tyr35. This is in contrast to dimethyl ether in 21J. (Fig. 3) Notably, both compounds can form a hydrogen bond with Asp168 through the nitrogen of the anilino group. This interaction is not observed in JNK3 structures (**Suppl. Figure 6**).

#### p38α:23GA vs. p38α:23M:

**Figure 5:**
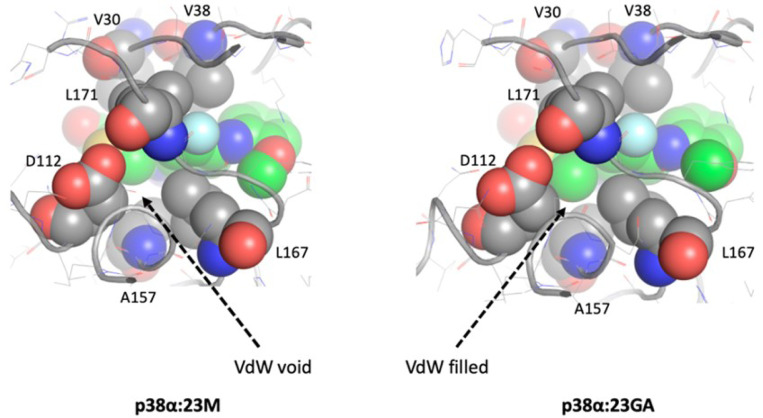
Comparison of the VdW interface between p38α:23M and p38α:23GA. The key residues surrounding the C4 of thiophene where methyl substitution occurs in 23G are shown in spheres. The VdW interface in the p38α:23GA complex is tightly filled compared to that of p38α:23M due to the addition of a methyl group, which enhances hydrophobic interaction.

Both complexes are in the DFG-out conformation. The methyl substitution at the thiophene in 23GA is surrounded by hydrophobic side chains from Ala 157, Leu167, Leu171, and Cβ of Asp112. The VdW crowding nudges 23GA towards the hinge compared to 23M. This minute movement, along with the rotation of the oxetane, brings the group closer to Arg49 to form a hydrogen bond (Fig. 5 **and Suppl. Figure 4**).

#### JNK3 vs. p38α

The involvement of the P-loop in ligand binding is more noticeable in p38α due to the presence of Tyr35, where a hydrophobic region of the ligand can stack with the tyrosyl ring. In contrast, the orthogonal residue to Tyr35 in JNK3 is Gln75, which is often disordered in crystal structures. Methyl substitution in thiophene pushes 23GA away from hydrophobic pocket I in both JNK3 and p38α (Fig. 2B and Fig. 5). While the substitution affects negatively for 23GA binding to JNK3, p38α benefits from the substitution in two ways; the hydrophobic addition sits in the region where multiple hydrophobic interaction partners are present, and it pushes the compound toward Arg49 which produces constructive H-bond interaction with the oxetane group of 23GA (**Suppl. Figure 4**). The residue corresponding to Arg49 in JNK3 is Asn89, which was not used for direct hydrogen bond interaction with a bound ligand. In p38α with 21G and 21J, Asp168 in the DFG-in state of p38α is held in place by Lys53 and can form H-bond interactions with the nitrogen of the anilino group. However, this interaction is absent in JNK3 due to differences in the conformation of the DFG residues (**Suppl. Figure 6**). The unmet need for an H-bond with 21G or 21J in JNK3 is satisfied by a water-mediated H-bond interaction through Lys93.

## DISCUSSION

The 6-anilino indazole scaffold was identified in the past as a lead for JNK3 selectivity [15]. The indazole scaffold exhibited higher brain penetration compared to the parent scaffold, which can be attributed to its rigidified structures and lower polar surface area (PSA). The derivative compounds act as ATP competitive inhibitors, occupying the ATP binding pocket at the cleft between the N- and C-lobe of the kinase. An SAR study conducted by Jiang *et al*. demonstrated that the scaffold can be modified to achieve selectivity for JNK3 over JNK1 and p38α [16]. The phenyl linker between the amide and indazole is crucial for both hinge binding and interaction with hydrophobic pocket II. Recent medicinal chemistry studies have used thiophene as a substitute for the phenyl group, and confirmed that this substitution was equally effective in hinge binding and hydrophobic interaction, which expanded the selection of chemotypes. Additionally, the use of thiophene allowed for further modification of the linkage. (e.g., methyl addition in 23GA). The crystal structures of JNK3 with the current probe compounds show that the interactions at the hinge binding site are consistent. Further analysis using MD simulations suggests that the H-bonding interaction through indazole nitrogen is optimal. However, the hinge binding interaction at amide nitrogen weakens when the bound ligand shifts (in the case of 23G) or completely breaks when the thiophene flips out.

Typically in a protein-ligand interaction, water molecules on the protein surface are displaced. Some water molecules may remain on the protein surface to satisfy the requirement for H-bond in ligand binding. If this interaction is enthalpically favorable, the water molecule can be substituted with a functional group in the ligand to enhance the binding affinity [17,18]. In the crystal structure of JNK3:21J, a stable water molecule was found to bridge between Asn152 and the dimethyl ether oxygen of 21J. This water bridge was also observed in the MD experiment for 34% of the simulation time. Based on these findings, Asn152 seems to be a good candidate for a H-bond partner for future inhibitor design.

The p38α structures exhibit two distinct inhibitor-induced conformational changes: DFG-out and a folded P-loop. In contrast, the JNK3 structures show only subtle changes in the P-loop conformation. For p38α structures, it is quite intriguing to observe that 23GA and 23M, with their relatively short hydrophobic moieties occupying the hydrophobic pocket I, are able to stabilize the DFG-out conformation without reaching into the DFG-out pocket. On the other hand, 21G and 21J prefer the DFG-in conformation. The DFG-in and -out states of p38α are in conformational equilibrium, as evidenced by NMR studies [19]. In the cases of 21G and 21J, it appears that an H-bond interaction between Asp186 and the N- of the anilino group prevents the DFG loop from flipping out. The methoxy group in 23GA and 23M precludes such interactions. There are no reports of a DFG-out structure for JNK3, while its isoform, JNK2, has one structure disclosed in DFG-out conformation with a diaryl urea p38α inhibitor, BIRB796 [20]. BIRB796 has high affinity for JNK2 (KD = 4.6 nM), but low affinity for JNK3 (KD = 62 nM), and no affinity for JNK1. A modeling and docking study by A. Messoussi et al. concluded that the binding characteristics of BIRB796 to JNK isoforms are determined by two residues near the hydrophobic pocket I (Met77 and Ile106 for JNK1; and Met115 for JNK3) [21]. Alternatively, it’s possible that JNK3 is locked in a DFG-in conformation, with the DFG-out conformation not being readily available for high-affinity binding with BIRB796. For example, Imatinib has been shown to bind to a number of kinases in a DFG-out conformation, while a covalent version of imatinib binds to JNK3 in a DFG-in conformation with a U-shaped folded conformation [22]. Therefore, it’s possible that BIRB796 also binds to JNK3 in a DFG-in conformation.

There are multiple differences in the hydrophobic interactions between JNK3 and p38α with the probe compounds. Two mutually exclusive conformations, the folded P-loop and DFG-out, strengthen hydrophobic interactions in p38α. The folded P-loop conformation is a unique feature in several kinases, including p38α, where the tip of the loop is either a Phe or a Tyr. The bound ligand drives loop folding through pi-stacking or robust hydrophobic interaction. It has been demonstrated that this folded conformation increases selectivity and potency without adding to the molecular weight of the ligand [23]. Such type of P-loop folding is unlikely to occur in JNK3 since the tip of the P-loop is a Gln residue. As we observed with 23GA, p38α favors the hydrophobic substitution at thiophene in the DFG-out conformation, where Leu171 provides an additional hydrophobic interaction (Fig. 5). In contrast, JNK3 disfavors this due to the presence of the polar residue Asn152. However, the overall hydrophobic interaction of the compounds with the pockets is tighter in JNK3 than in p38α, primarily due to bulkier hydrophobic residues Val196 and Met146 compared to the equivalent residues Ala157 and Thr106 in p38α (**Suppl. Figure 7**).

## CONCLUSION

In this study, we determined the crystal structures of JNK3 and p38α in complex with four indazole scaffold compounds that exhibit selectivity either for JNK3 or p38α. We also investigated the determinants for selectivity within both kinases. Aside from the gatekeeper residues that control access to the hydrophobic site I, the inhibitors utilize various degrees of all available binding mechanisms for selectivity; these include hydrophobic pocket I, the P-loop, the hinge residue, and hydrophobic pocket II with peripheral residues. We believe this study was a useful exercise in understanding the selectivity mechanism of the indazole scaffold. More importantly, this study serves as a comprehensive guide for designing new JNK3 selective inhibitors.

Some of the key findings from this study include: 1) in the crystal structure of JNK3:21J, a stable water molecule was observed, which bridges between Asn152 and the dimethyl ether oxygen of 21J and this water molecule provides an enthalpic advantage and can enhance the binding affinity, 2) methyl substitution at the thiophene ring is favored in p38α due to the presence of surrounding hydrophobic residues, whereas the opposite is true for JNK3, 3) shifts in ligand binding in JNK3 and p38α have opposite effects on their affinity, 4) JNK3 prefers the DFG-in conformation, whereas p38α exhibits a conformational equilibrium between DFG-in and DFG-out states.

The study has limitations in that it relied solely on X-ray crystallography and simulation methods to investigate kinase-ligand binding. To further validate the key observations, additional experimental tools such as NMR, Hydrogen-Deuterium Exchange Mass Spectrometry (HDX-MS), and Isothermal Titration Calorimetry (ITC) should be employed to empirically observe dynamics and binding. These tools would provide complementary information on protein-ligand interactions, stability, dynamics, and binding affinities, thereby enhancing the understanding and confidence in the findings presented in the study.

## EXPERIMENTAL

### Synthesis and characterization of compounds 21G, 21J, 23GA, and 23M.

The four compounds were prepared following similar synthetic routes as previously described [9].

#### 21G.

^1^H NMR (CD_3_OD, 400 MHz): δ (ppm) 8.11 (s, 1H), 8.05 (s, 1H), 7.94 (d, *J* = 1.6 Hz, 1H), 7.38–7.35 (m, 1H), 7.22–7.14 (m, 3H), 4.82 (s, 2H), 4.31–4.22 (m, 4H), 2.96 (s, 3H), 2.61–2.49 (m, 2H). Analytical LC, one major peak with purity of 98.2% based on UV absorption at 254 nm. Chemical Formula: C23H21ClFN5OS. Mass spectroscopy (ESI), calculated for [M + H] = 470.1, observed: [M + H] = 469.6.

#### 21J.

^1^H NMR (DMSO_d_6_, 400 MHz): δ (ppm) 8.72–8.71 (m, 1H), 8.22 (s, 1H), 8.15 (s, 1H), 8.01 (s, 1H), 7.93 (s, 1H), 7.45–7.43 (d, *J* = 8.4 Hz, 1H), 7.37 (t, *J* = 9.6 Hz, 1H), 7.23–7.20 (m, 1H), 7.15 (m, 1H), 6.85–6.84 (m, 1H), 4.75 (s, 2H), 3.40 (s, 3H), 2.81–2.80 (m, 3H). Analytical LC, one major peak with purity of 96.7% based on UV absorption at 254 nm. Chemical Formula: C21H18ClFN4O2S. Mass spectroscopy (ESI), calculated for [M + H] = 445.1, observed: [M + H] = 444.5.

#### 23GA.

1H NMR (400 MHz, *DMSO-d6*) δ 9.20 (d, *J* = 6.4 Hz, 1H), 8.28 (s, 1H), 8.00 (s, 1H), 7.59 (d, *J* = 8.0 Hz, 1H), 7.43 (d, *J* = 11.2 Hz, 1H), 7.05–6.99 (m, 2H), 6.89–6.80 (m, 3H), 5.04–4.95 (m, 1H), 4.79 (t, *J* = 7.2 Hz, 2H), 4.58 (t, *J* = 6.4 Hz, 2H), 3.85 (s, 3H), 2.41 (s, 3H). Analytical LC, one major peak with purity of 99.2% based on UV absorption at 254 nm. Chemical Formula: C23H21FN4O3S. Mass spectroscopy (ESI), calculated for [M + H] = 453.1, observed: [M + H] = 453.0.

#### 23M

1H NMR (400 MHz, DMSO-d6) δ 9.40 (d, J = 6.4 Hz, 1H), 8.40 (s, 1H), 8.28 (s, 1H), 8.09 (s, 1H), 7.93 (d, J = 11.2 Hz, 1H), 7.57 (d, J = 8.0 Hz, 1H), 7.09 (s, 1H), 7.03 (d, J = 7.6 Hz, 1H), 6.94–6.80 (m, 3H), 5.01 (m, 1H), 4.81 (t, J = 6.8 Hz, 2H), 4.62 (t, J = 6.2 Hz, 2H), 3.85 (s, 3H). Analytical LC, one major peak with purity of 97.9% based on UV absorption at 254 nm. Chemical Formula: C22H19FN4O3S. Mass spectroscopy (ESI), calculated for [M + H] = 439.1, observed: [M + H] = 438.9.

### Homogeneous Time-resolved Fluorescence Assay (HTRF)

Enzyme inhibition assays to determine IC_50_ values for the kinases were performed as described previously [7–9,24].

### Preparation of human JNK3 and mouse p38α, and crystallization

Purification of JNK3 39–402 and its crystallization with ATP was done following a previously published procedure ([7–9,24]. The JNK3 inhibitors were soaked into JNK3-ATP crystals by incubating the crystals overnight in a reservoir solution containing 2 mM of each compound. Cloning, expression, and purification of p38α were done following the report published previously with variations [25]. Briefly, the cDNA of mouse p38α was obtained from the inhouse mouse ORF complete collection, amplified by PCR and cloned into *NdeI*/*BamHI* sites of pET24b vector (Novagen) to produce full-length p38α with an N-terminal 6-histidine tag. The plasmid was transformed into the *E. coli* Rosetta 2 strain (Novagen) and expressed for 3 hrs at 22°C using LB media that was supplemented with kanamycin and Chloramphenicol. Purification of p38α was done by two-step purification of nickel affinity column and size exclusion chromatography (SEC) column on an Akta FPLC (GE Healthcare). Bacterial culture was harvested by centrifugation and sonicated after resuspending in a buffer containing 500 mM NaCl, 10 mM imidazole, 10 mM Tris-HCl, pH 8.0, and 10% glycerol. Supernatant after sonication was loaded onto a 2 mL His-Select column (Sigma). The column was washed with 30 mL of the sonication buffer to remove impurities. p38α was eluted with a linear gradient from 10 to 500 mM imidazole. The eluted peak fractions were pooled and concentrated to around 5 mL using a 30-kDa cut off ultrafiltration unit (Millipore). The concentrated eluent was loaded onto Superdex 200 26/60 SEC column that was pre-equilibrated with a buffer containing 25 mM Tris–HCl, pH 7.5, 100 mM NaCl, 10 mM MgCl2, 10mM DTT, and 5% glycerol. The fractions containing pure p38α were pooled and concentrated at 14 mg/ml. For crystallization, an equal volume of p38α was mixed with the reservoir solution containing 14% PEG 4K, M Na cacodylate, pH 6.5, 10 mM n-octyl-b-D-glucoside (b-OG), and 1 mM ATP, and incubated in hanging drop plates. The compounds were soaked into p38α-ATP crystals by adding 2 mM of each compound in reservoir solution into a crystallization drop and incubating for 24 hrs.

### Data collection and structure determinations

JNK3 crystals were transferred to mounting loops and flash-frozen in liquid nitrogen after removing excess drop solutions. p38α crystals were cryoprotected in a reservoir solution that is supplemented with 20% ethylene glycol and flash-frozen in liquid nitrogen. The data collections were done on Pilatus 6M at SSRL beamline 12 – 2. The datasets were processed with iMOSFLM (CCP4 suite) [26,27]. JNK3 and p38α crystals were phased using Phaser (Phenix suite) [28] with PDB ID 1JNK and 4LOO, respectively, as the search model for molecular replacements. All phased maps showed positive densities for the target compounds in the ATP pocket. Restraints and coordinates for the compounds were generated using eLBOW (Phenix suite) [28] and incorporated into protein coordinates. The models were refined using phenix.refine (Phenix suite) [28]. The models were manually inspected and adjusted after each refinement cycle using Coot [29]. Data processing and refinement statistics are given in **Supplemental Tables 1 and 2**. The final coordinates were deposited with PDB IDs 8VO4 (JNK3:21G), 8VNX(JNK3:21J), 8VS0 (JNK3:23GA), 8VTF (JNK3:23M), 8VWM (p38α:21G), 8VT6 (p38α:21J), 8VMH (p38α:23GA), and 8VMM (p38α:23M)

### Coordinate regularization

For proper compound binding analysis of the structures, the refined coordinates from X-ray crystallography were minimized in Schrodinger Suite release 2022–1. Briefly, the structures were prepared by assigning the bond order and repairing missing side chains and hydrogen atoms. pK values of charged side chains were calculated using PROPKA at pH 7. Finally, water orientations were corrected and the whole systems were minimized using OPLS4 force field [30] while the heavy atoms are restrained to RMSD of 0.3 Å. The prepared structures were further minimized using Prime (Schrodinger, Inc). Structural analysis was done with PyMol (Schrodinger, Inc) and Maestro (Schrodinger, Inc)

### MD simulation

Missing loops were modeled and minimized in Prime (Schrodinger, Inc). The structures were neutralized and solvated with 150 mM KCl, TIP3P water molecules, and counter ions in an orthorhombic box with a 10 Å buffer in each direction. The OPLS4 force field [30] was used in all simulations. After the initial 20 ns minimization with the isothermal and isobaric ensemble, the systems were relaxed and ran for 0.5 µs simulations. The kinase-ligand interaction analyses of the results were done in the Maestro simulation analysis tool (Schrodinger, Inc). The initial 0.1 µs simulations were removed from the analysis.

## Figures and Tables

**Table 1:** The probe compound structures and their IC_50_ values determined by inhibition assays.

Compound	Compound structure	JNK3 (μM)	p38α (μM)
**21G**	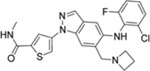	0.137	0.01
**21J**	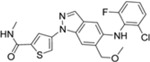	0.006	0.21
**23GA**	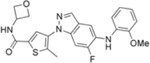	1	0.032
**23M**	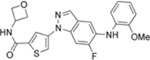	0.014	0.122

## Data Availability

The datasets generated and analyzed during the current study are available in the PDB repository, https://www.rcsb.org/.

## References

[1] Ly K.C.D., Peterson J.R., The Human Kinome and Kinase Inhibition, Current Protocols in Pharmacology 60 (2013) 2.9.1–2.9.14. 10.1002/0471141755.ph0209s60. PMC4128285 23456613

[2] Davis M.I., Hunt J.P., Herrgard S., Ciceri P., Wodicka L.M., Pallares G., Hocker M., Treiber D.K., Zarrinkar P.P., Comprehensive analysis of kinase inhibitor selectivity, Nat Biotechnol 29 (2014) 1046–1051. 10.1038/nbt.1990. 22037378

[3] Antoniou X., Falconi M., Marino D.D., Borsello T., JNK3 as a Therapeutic Target for Neurodegenerative Diseases, J Alzheimer’s Dis 24 (2011) 633–642. 10.3233/jad-2011-091567. 21321401

[4] Rehfeldt S.C.H., Majolo F., Goettert M.I., Laufer S., c-Jun N-Terminal Kinase Inhibitors as Potential Leads for New Therapeutics for Alzheimer’s Diseases., IJMS 21 (2020) 9677. 10.3390/ijms21249677. 33352989 PMC7765872

[5] Yarza R., Vela S., Solas M., Ramirez M.J., c-Jun N-terminal Kinase (JNK) Signaling as a Therapeutic Target for Alzheimer’s Disease, Front Pharmacol 6 (2016) 321. 10.3389/fphar.2015.00321. 26793112 PMC4709475

[6] Canovas B., Nebreda A.R., Diversity and versatility of p38 kinase signalling in health and disease, Nature Reviews Molecular Cell Biology (2021) 1–21. 10.1038/s41580-020-00322-w. 33504982 PMC7838852

[7] Park H., Iqbal S., Hernandez P., Mora R., Zheng K., Feng Y., LoGrasso P., Structural Basis and Biological Consequences for JNK2/3 Isoform Selective Aminopyrazoles, Sci Rep-Uk 5 (2015) 8047. 10.1038/srep08047. PMC4306959 25623238

[8] Feng Y., Park H., Ryu J.C., Yoon S.O., N-Aromatic-Substituted Indazole Derivatives as Brain-Penetrant and Orally Bioavailable JNK3 Inhibitors, Acs Med Chem Lett 12 (2021) 1546–1552. 10.1021/acsmedchemlett.1c00334. 34676036 PMC8521607

[9] Feng Y., Park H., Bauer L., Ryu J.C., Yoon S.O., Thiophene-Pyrazolourea Derivatives as Potent, Orally Bioavailable, and Isoform-Selective JNK3 Inhibitors, Acs Med Chem Lett 12 (2020) 24–29. 10.1021/acsmedchemlett.0c00533. 33488960 PMC7812606

[10] Zheng K., Iqbal S., Hernandez P., Park H., LoGrasso P.V., Feng Y., Design and Synthesis of Highly Potent and Isoform Selective JNK3 Inhibitors: SAR Studies on Aminopyrazole Derivatives, J Med Chem 57 (2014) 10013–10030. 10.1021/jm501256y. 25393557 PMC4266361

[11] Zheng K., Park C.M., Iqbal S., Hernandez P., Park H., LoGrasso P.V., Feng Y., Pyridopyrimidinone Derivatives as Potent and Selective c-Jun N-Terminal Kinase (JNK) Inhibitors, Acs Med Chem Lett 6 (2015) 413–418. 10.1021/ml500474d. 25893042 PMC4394340

[12] Durrant J.D., McCammon J.A., Molecular dynamics simulations and drug discovery, Bmc Biol 9 (2011) 71–71. 10.1186/1741-7007-9-71. 22035460 PMC3203851

[13] Hollingsworth S.A., Dror R.O., Molecular Dynamics Simulation for All, Neuron 99 (2018) 1129–1143. 10.1016/j.neuron.2018.08.011. 30236283 PMC6209097

[14] Diskin R., Engelberg D., Livnah O., A Novel Lipid Binding Site Formed by the MAP Kinase Insert in p38α, J. Mol. Biol. 375 (2008) 70–79. 10.1016/j.jmb.2007.09.002. 17999933

[15] Swahn B.-M., Huerta F., Kallin E., Malmström J., Weigelt T., Viklund J., Womack P., Xue Y., Öhberg L., Design and synthesis of 6-anilinoindazoles as selective inhibitors of c-Jun N-terminal kinase-3, Bioorg Med Chem Lett 15 (2005) 5095–5099. 10.1016/j.bmcl.2005.06.083. 16140012

[16] Jiang R., Frackowiak B., Shin Y., Song X., Chen W., Lin L., Cameron M.D., Duckett D.R., Kamenecka T.M., Design and synthesis of 1-aryl-5-anilinoindazoles as c-Jun N-terminal kinase inhibitors, Bioorg Med Chem Lett 23 (2013) 2683–2687. 10.1016/j.bmcl.2013.02.082. 23518277

[17] Matricon P., Suresh R.R., Gao Z.-G., Panel N., Jacobson K.A., Carlsson J., Ligand design by targeting a binding site water, Chem Sci 12 (2020) 960–968. 10.1039/d0sc04938g. 34163862 PMC8179138

[18] Maurer M., Oostenbrink C., Water in protein hydration and ligand recognition, J Mol Recognit 32 (2019) e2810. 10.1002/jmr.2810. 31456282 PMC6899928

[19] Vogtherr M., Saxena K., Hoelder S., Grimme S., Betz M., Schieborr U., Pescatore B., Robin M., Delarbre L., Langer T., Wendt K.U., Schwalbe H., NMR Characterization of Kinase p38 Dynamics in Free and Ligand-Bound Forms, Angewandte Chemie Int Ed 45 (2006) 993–997. 10.1002/anie.200502770. 16374788

[20] Kuglstatter A., Ghate M., Tsing S., Villaseñor A.G., Shaw D., Barnett J.W., Browner M.F., X-ray crystal structure of JNK2 complexed with the p38α inhibitor BIRB796: Insights into the rational design of DFG-out binding MAP kinase inhibitors, BIOORGANIC & MEDICINAL CHEMISTRY LETTERS 20 (2010) 5217–5220. 10.1016/j.bmcl.2010.06.157. 20655210

[21] Messoussi A., Chevé G., Bougrin K., Yasri A., Insight into the selective inhibition of JNK family members through structure-based drug design, Medchemcomm 7 (2016) 686–692. 10.1039/c5md00562k.

[22] Zhang T., Inesta-Vaquera F., Niepel M., Zhang J., Ficarro S.B., Machleidt T., Xie T., Marto J.A., Kim N., Sim T., Laughlin J.D., Park H., LoGrasso P.V., Patricelli M., Nomanbhoy T.K., Sorger P.K., Alessi D.R., Gray N.S., Discovery of Potent and Selective Covalent Inhibitors of JNK, Chem Biol 19 (2012) 140–154. 10.1016/j.chembiol.2011.11.010. 22284361 PMC3270411

[23] Guimarães C.R.W., Rai B.K., Munchhof M.J., Liu S., Wang J., Bhattacharya S.K., Buckbinder L., Understanding the Impact of the P-loop Conformation on Kinase Selectivity, J Chem Inf Model 51 (2011) 1199–1204. 10.1021/ci200153c. 21568278

[24] Kamenecka T., Habel J., Duckett D., Chen W., Ling Y.Y., Frackowiak B., Jiang R., Shin Y., Song X., LoGrasso P., Structure-activity relationships and X-ray structures describing the selectivity of aminopyrazole inhibitors for c-Jun N-terminal kinase 3 (JNK3) over p38., J Biol Chem 284 (2009) 12853–12861. 10.1074/jbc.m809430200. 19261605 PMC2676016

[25] Bukhtiyarova M., Northrop K., Chai X., Casper D., Karpusas M., Springman E., Improved expression, purification, and crystallization of p38α MAP kinase, Protein Expression and Purification 37 (2004) 154–161. 10.1016/j.pep.2004.05.017. 15294293

[26] Battye T.G.G., Kontogiannis L., Johnson O., Powell H.R., Leslie A.G.W., iMOSFLM : a new graphical interface for diffraction-image processing with MOSFLM, Acta Crystallogr. Sect. D Biol. Crystallogr. 67 (2011) 271–281. 10.1107/s0907444910048675. 21460445 PMC3069742

[27] Winn M.D., Ballard C.C., Cowtan K.D., Dodson E.J., Emsley P., Evans P.R., Keegan R.M., Krissinel E.B., Leslie A.G.W., McCoy A., McNicholas S.J., Murshudov G.N., Pannu N.S., Potterton E.A., Powell H.R., Read R.J., Vagin A., Wilson K.S., Overview of the CCP4 suite and current developments, Acta Crystallogr. Sect. D 67 (2011) 235–242. 10.1107/s0907444910045749. 21460441 PMC3069738

[28] Adams P.D., Grosse-Kunstleve R.W., Hung L.W., Ioerger T.R., McCoy A.J., Moriarty N.W., Read R.J., Sacchettini J.C., Sauter N.K., Terwilliger T.C., PHENIX: building new software for automated crystallographic structure determination., Acta Crystallogr. Sect. D, Biol. Crystallogr. 58 (2002) 1948–54. 10.1107/s0907444902016657. 12393927

[29] Emsley P., Lohkamp B., Scott W.G., Cowtan K., Features and development of Coot, Acta Crystallogr. Sect. D 66 (2010) 486–501. 10.1107/s0907444910007493. 20383002 PMC2852313

[30] Lu C., Wu C., Ghoreishi D., Chen W., Wang L., Damm W., Ross G.A., Dahlgren M.K., Russell E., Bargen C.D.V., Abel R., Friesner R.A., Harder E.D., OPLS4: Improving Force Field Accuracy on Challenging Regimes of Chemical Space, J. Chem. Theory Comput. 17 (2021) 4291–4300. 10.1021/acs.jctc.1c00302. 34096718

